# Timely Surgical Management of a Large Cervical Fibroid Presenting With Urinary Retention

**DOI:** 10.7759/cureus.88830

**Published:** 2025-07-26

**Authors:** Gulnaaz Khan, Varsha Kose

**Affiliations:** 1 Obstetrics and Gynecology, Narendra Kumar Prasadrao (NKP) Salve Institute of Medical Sciences and Research Centre, Nagpur, IND; 2 Obstetrics and Gynecology, Narendra Kumar Prasadrao (NKP) Salve Institute of Medical Sciences and Research Centre, Lata Mangeshkar Hospital, Nagpur, IND

**Keywords:** cervical fibroid, obstructive uropathy, total abdominal hysterectomy, urinary retention, uterine leiomyomas

## Abstract

Cervical fibroids are rare and account for a small percentage of uterine leiomyomas. A 34-year-old woman visited the department of obstetrics and gynaecology with chief complaints of fever with chills and burning micturition for three days. She also complained of urinary retention for one month, along with post-coital and intermenstrual bleeding. On per vaginal examination, a large pelvic mass filling the vagina, approximately equivalent to a 15-16 weeks gravid uterus, was observed, and a provisional diagnosis of abnormal uterine bleeding (AUB-L) due to a cervical fibroid was made. The diagnosis was confirmed by transabdominal ultrasonography (USG) and magnetic resonance imaging (MRI), which demonstrated a large anterior cervical fibroid. The surgical intervention involved total abdominal hysterectomy (TAH) with bilateral salpingectomy, wherein a large cervical fibroid was identified and removed. The surgical procedure was reported as technically challenging due to the anatomical distortion caused by the fibroid. The postoperative course was uneventful, and the patient reported complete resolution of urinary symptoms at a 15-day follow-up. This case highlights the importance of early diagnosis and surgical planning in managing large cervical fibroids to avoid complications such as obstructive uropathy and surgical injuries.

## Introduction

Leiomyomas are benign smooth muscle tumors of the uterus, which are the most frequent pelvic tumors in women of reproductive age [1]. Cervical fibroids represent less than 1-2% of all uterine fibroids. The cervical fibroids are classified as intracervical or extracervical on the basis of location and could be present in anterior, posterior, lateral, or central positions [2]. Large cervical fibroids may cause pressure symptoms such as urinary retention and pose significant challenges during surgical excision due to the proximity of critical pelvic structures like the bladder, rectum, and ureters [3,4]. Prompt diagnosis and appropriate surgical management are vital to improve quality of life.

## Case presentation

Patient information

A 34-year-old woman reported to the obstetrics and gynecology department with the chief complaints of fever, chills, and burning micturition for three days, along with urinary retention, intermenstrual and post-coital bleeding persisting for one month. The obstetric history reported that the patient had two pregnancies delivered following spontaneous vaginal deliveries, resulting in two living children (P2L2). The menstrual history revealed a regular cycle with four to five days of moderate flow. The medical history reported a psychiatric disorder (hallucinations) six years ago, for which she took aripiprazole, an atypical antipsychotic drug, for five years and stopped for one year.

Clinical examination

The patient presented with stable vitals except mild pallor. On per abdominal examination, a soft, non-tender abdomen with a suprapubic palpable mass was revealed. On per speculum examination, the cervix could not be visualized, and the pink mass was protruding within the vagina. On per vaginal examination, a large mass occupying the entire vaginal canal, obscuring the cervix, and equivalent to a 15 to 16-week gestational uterus on bimanual exam had been noticed. A provisional diagnosis of abnormal uterine bleeding (AUB-L) due to a cervical fibroid with a FIGO score of eight was made.

Diagnostic assessment

**Figure 1:**
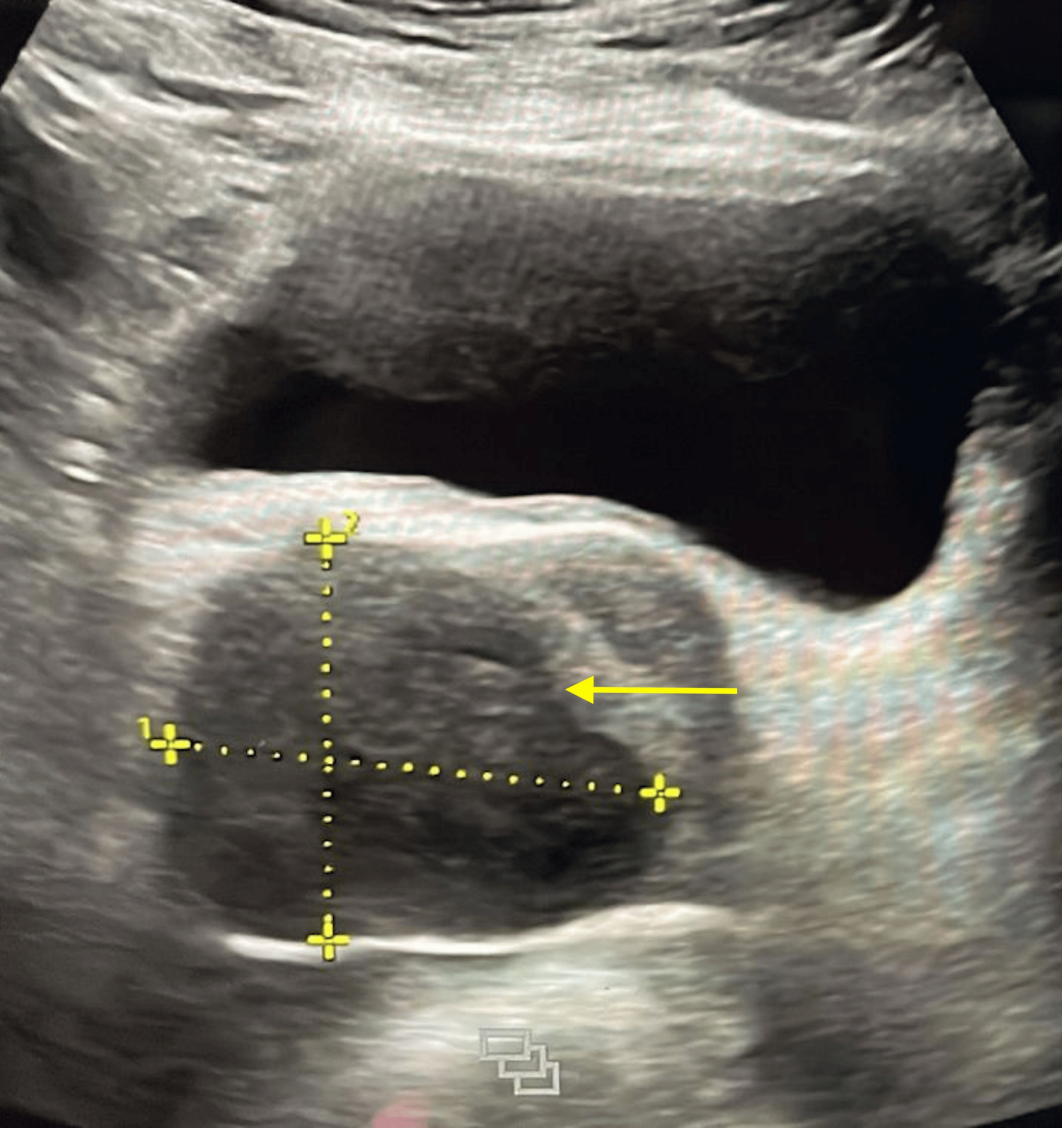
Transabdominal ultrasonography (USG) pointing with yellow arrow at a large well-defined heterogeneous hypoechoic lesion in the anterior wall of the lower uterine segment

For diagnostic assessment, the transabdominal ultrasonography (USG) was performed, which revealed a large, well-defined heterogeneous hypoechoic lesion (10.3×8.1×10 cm) in the anterior wall of the lower uterine segment (subserosal) as shown in Figure 1 with minimal peripheral vascularity on color Doppler.

**Figure 2:**
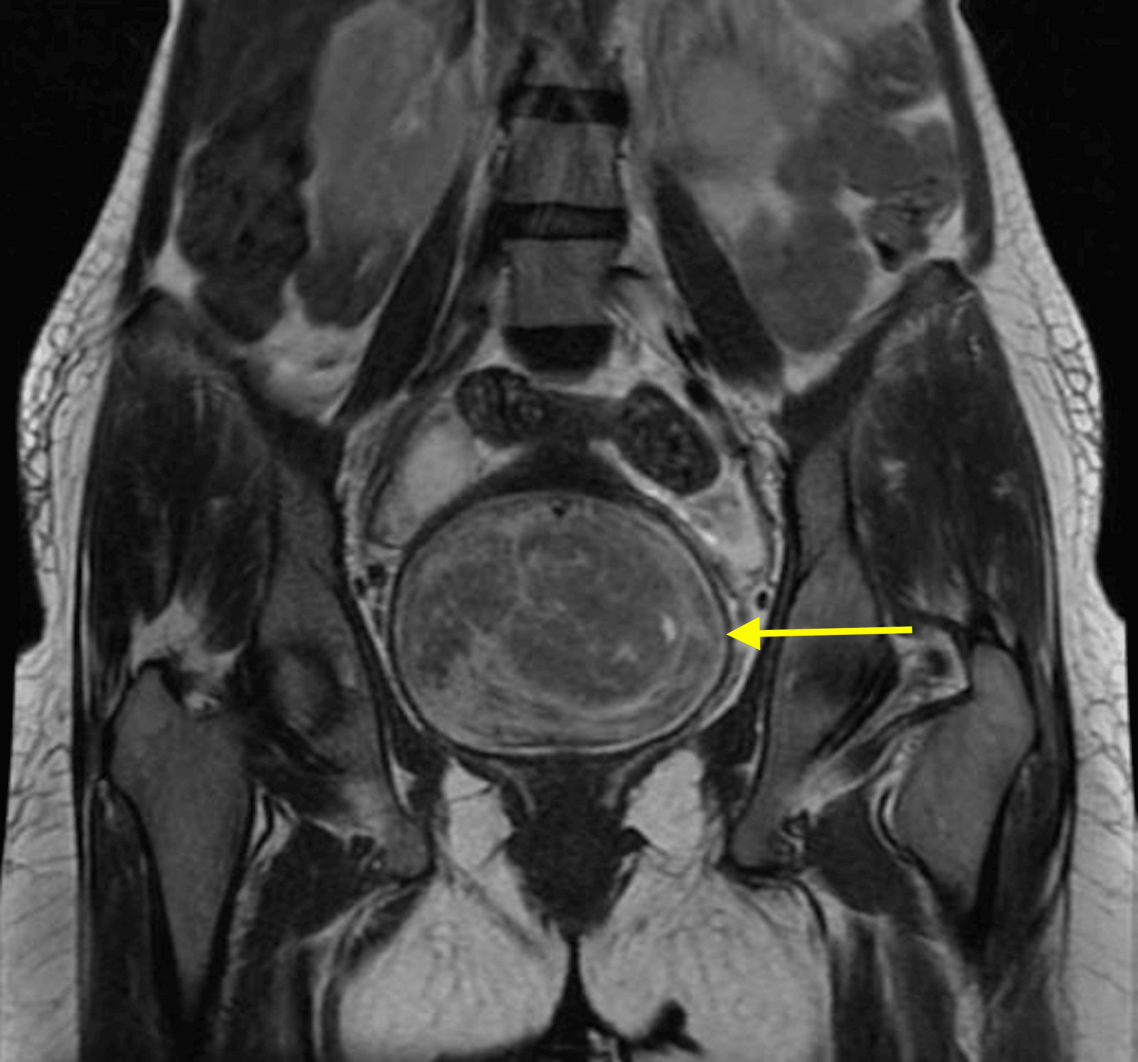
Magnetic resonance imaging (MRI) with yellow arrow pointing at a large well defined heterogeneous altered signal intensity lesion measuring 11.5 × 9.5 × 9.9 cm

The magnetic resonance imaging (MRI) revealed a large, well-defined heterogeneous altered signal intensity lesion measuring 11.5 × 9.5 × 9.9 cm epicentred in the pelvis, likely arising from the anterior lip of the cervix, and is noted occupying extending from level of S1 vertebra up to the coccygeal vertebra with non-enhancing cystic areas within, as shown in Figure 2.

The urology consultation was conducted following the chief complaints of the patient. The urodynamic test revealed that the bladder compliance was normal, the maximum cystometric capacity was within the normal range of 430 ml, with no detrusor overactivity. An elevated detrusor pressure of 62 cm H₂O was noted, which suggested obstruction in outflow. A significant reduction in maximum urinary flow rate, 7 ml/sec, was noted, and an elevated post-void residual of 180 ml was noted. Also, an increased urethral closure pressure in the mid-urethra, along with prolonged detrusor contraction with poor flow in the voiding phase, were noted, which indicated mechanical obstruction. These findings confirmed that the urinary retention was due to bladder outlet obstruction secondary to mass effect from the anterior cervical fibroid compressing the urethra.

Therapeutic intervention

The patient was counselled, and after pre-anesthetic clearance, the patient underwent stenting of the ureters followed by total abdominal hysterectomy (TAH) with bilateral salpingectomy. The stenting of the ureters was done to prevent the risk of ureteral injury during surgery. Intraoperatively, a large anterior cervical fibroid was encountered. Vasopressin infiltration was used to minimize bleeding. A myoma screw and coring technique was utilized to remove the fibroid, with part of the specimen excised as a whole. The patient received one unit of packed red cells intraoperatively. The procedure was technically challenging due to the anatomical distortion and proximity to critical structures. However, careful dissection and use of intraoperative techniques such as vasopressin infiltration and uterine manipulation aided in successful removal. The histopathological examination (HPE) confirmed cervical leiomyoma with chronic cervicitis. The recovery was uneventful, and the patient was discharged in stable condition on the ninth day post-surgery. She voided spontaneously with a normal stream, and no post-void residual urine on follow-up ultrasound was noted. At a 15-day follow-up, she reported complete resolution of urinary symptoms, including frequency, urgency, and retention.

## Discussion

Cervical fibroids, though rare, may cause significant morbidity due to pressure effects and anatomical distortion [5]. It often presents with abnormal bleeding, pelvic pain, urinary symptoms, or, rarely, urinary retention as in the present case [4]. Diagnosis is primarily imaging-based, with USG as the first-line modality and MRI for precise localization and surgical planning [6].

The therapeutic intervention involved surgery, depending upon the patient's age and childbearing status, either hysterectomy or myomectomy [7]. In young women, especially those desiring fertility preservation, conservative surgical approaches such as cervical myomectomy or uterine artery embolization (UAE) may be considered [7,8]. However, these options are feasible only for smaller, well-delineated fibroids that are not causing severe anatomical distortion or hydronephrosis. In this case, the size and position of the fibroid posed significant challenges, and hysterectomy offered a definitive solution.

There can be surgical difficulties while operating due to the anatomic correlation, having the bladder anteriorly, rectum posteriorly, and the bilateral ureters lateral to the cervical fibroid. The size of the fibroid alters the position of these anatomical structures, and there always remains the risk of injury and hemorrhage, so this surgery requires an experienced surgeon [3,4]. Bleeding can be prevented with dilute vasopressin, bilateral uterine artery ligation, use of a vessel clip temporarily, and an internal iliac balloon occlusion catheter. UAE or uterine fibroid embolization can be done under interventional radiology [9].

Ureteric injury remains the most dreaded complication of the surgery. Therefore, stenting of the ureters before the surgery could reduce the risk of ureteric injury, which helps to recognize the ureters easily intraoperatively. So, careful dissection should be done. Intraoperatively, uterine patency should be checked [3,10]. In this case, the ureters were patent. Patency was checked with the methylene blue dye technique. If injury occurs, ureteric stenting should be done with the assistance of a urosurgeon.

The distorted pelvic anatomy makes surgery challenging, with increased risks of hemorrhage and injury to the bladder, ureters, or bowel [11]. Careful surgical planning, use of vasopressin to minimize bleeding, and intraoperative identification of ureters (aided by methylene blue or stenting if necessary) are essential. In the present case, successful surgery and resolution of symptoms were achieved without complications, emphasizing the importance of expertise and preoperative planning. However, it is important to consider the long-term quality of life outcomes following hysterectomy. Studies have shown that sexual function can be preserved or even improved in many women post-hysterectomy [12]; however, risks such as vaginal vault prolapse, pelvic floor dysfunction, or psychological impact related to loss of fertility must be discussed preoperatively [7].

## Conclusions

Large cervical fibroids are rare but can lead to significant pressure symptoms, including urinary retention. Accurate diagnosis with imaging and timely surgical management are crucial to prevent complications such as obstructive uropathy or organ injury. This case specifies the surgical complexity associated with cervical fibroids and the importance of a skilled multidisciplinary approach for optimal outcomes. Also, it recommends including urodynamic evaluation when urinary symptoms are involved to understand the impact on the lower urinary tract. Furthermore, it recommends considering conservative approaches like myomectomy or UAE in appropriate candidates and to counsel patients regarding the long-term impact on sexual function and prolapse risk, to support informed decision-making.
